# Digital educational technology on LGBT-phobic bullying for school-aged adolescents

**DOI:** 10.1590/0034-7167-2024-0377

**Published:** 2025-09-08

**Authors:** Mariana Mercês Mesquita Espíndola, Ednaldo Cavalcante de Araújo, Adrian Thaís Cardoso Santos Gomes da Silva, Danilo Martins Roque Pereira, Marclineide Nóbrega de Andrade Ramalho, Thainara Torres de Oliveira, Weslla Karla Albuquerque Silva de Paula, Josueida de Carvalho Sousa

**Affiliations:** I Universidade Federal de Pernambuco. Recife, Pernambuco, Brazil; II Universidade Católica de Pernambuco. Recife, Pernambuco, Brazil

**Keywords:** Adolescent, Bullying, Sexual and Gender Minorities, Comic Book, Educational Technology, Bullying, Minorias Sexuales y de Género, Libro de Historietas, Tecnología Educacional

## Abstract

**Objectives:**

to develop a digital educational technology on LGBT-phobic bullying, in the form of a comic book, for health education among school-aged adolescents.

**Methods:**

a methodological study employing the Planning of Computer-Supported Learning Activities method to guide the organization of development stages, combined with Edgar Morin’s pedagogical framework, under the perspective of comprehension, health education, and the context of sexual and gender diversity.

**Results:**

the comic book “LGBT-Phobic Bullying: Shall We Talk?” was developed with the aim of contributing to education and awareness in the fight against LGBT-phobic bullying in school environments, serving as a health educational technology product.

**Final Considerations:**

as a contemporary approach, this technology was designed to address a sensitive topic in adolescent LGBTQIAP+ health in a reflective manner. The combination of visual storytelling in comics with educational messages can effectively contribute, alongside educational practices, to reducing stigma, prejudice, bullying, and other forms of violence against LGBTQIAP+ adolescents.

## RESUMEN

**Objetivos:**

desarrollar una tecnologia educativa digital sobre el acoso LGBTIfóbico, en formato de cómic, para la educación en salud de adolescentes escolares.

**Métodos:**

estudio metodológico que emplea el método de Planificación de Actividades de Aprendizaje Apoyadas por Computadora para guiar la organización de las etapas de desarrollo, combinado con el marco pedagógico de Edgar Morin, bajo la perspectiva de la comprensión, la educación en salud y el contexto de la diversidad sexual y de género.

**Resultados:**

el cómic “Acoso LGBTIfóbico: ¿Hablemos?” fue desarrollado con el propósito de contribuir a la educación y concienciación en la lucha contra el acoso LGBTIfóbico en el entorno escolar, constituyéndose como un producto de tecnologia educativa en salud.

**Consideraciones Finales:**

como una aproximación contemporânea, esta tecnologia fue diseñada para abordar de manera reflexiva un tema sensible en la salud de los adolescentes LGBTQIAP+. La combinación del poder narrativo-visual de los cómics con mensajes educativos puede contribuir eficazmente, junto con las prácticas educativas, a la reducción del estigma, el prejuicio, el acoso y otras formas de violencia contra adolescentes LGBTQIAP+.

## INTRODUCTION

Lesbian, gay, bisexual, transgender, queer/questioning, intersex, asexual/aromantic/agender, poly/pansexual, and other individuals belonging to the spectrum of sexual and gender diversity (LGBTQ-IAP+) are, for the most part, in contexts of vulnerability, where they are routinely subjected to bullying, stigma, and verbal, psychological, and/or physical violence, which can lead to suicide and even murder perpetrated by the aggressor. Additionally, they experience social discrimination, which affects their quality of life and psychological well-being, leading to negative emotions, symptoms of anxiety, and other health-compromising behaviors^(1, 2, 3)^.

Characterized by physical, psycho-emotional, and social transformations, as well as by self-questioning and feelings of self-acceptance, adolescence and youth are stages in which behaviors and attitudes are developed, and habits and values are formed, influencing the well-being and development of these individuals^(4, 5, 6)^. In this regard, discussions about the expression of sexuality during this period are essential for understanding lived experiences. However, this topic often becomes taboo, with discussions marked by superficiality, prejudice, and discomfort. This occurs to the detriment of pseudo-normative scenarios that, at times, delegitimize experiences that should be recognized as fundamental and integral components of human life^(7, 8)^.

Gender identity is defined as an individual’s intrinsic perception of themselves as female, male, or a combination of both, which may or may not align with their biological sex. Gender expression refers to how a person presents themselves through clothing, speech patterns, body language, and social interactions^(9, 10)^.

The term “trans” serves as an umbrella term in Brazil to refer to people who identify as travestis, transgender women, transgender men, transmasculine or transfeminine individuals, non-binary individuals, and others with diverse gender identities. For transgender adolescents, the challenges they face begin with the construction of their gender identity and extend to family acceptance and socialization in school environments. When stigmatized, these adolescents experience exclusion, discrimination, LGBT-phobic bullying, insecurity, and negative attitudes that reinforce violent contexts^(2, 11, 12, 13, 14, 15)^.

That said, school bullying is recognized as a form of violence and represents a global and complex phenomenon in contemporary society. It occurs in interpersonal relationships and is structured as a construct of domination influenced by various factors ‘individual, social, emotional, psychological, contextual, etc.’. It is directly correlated with symptoms of depression, anxiety, stress, and suicidal ideation, challenging the misconception that bullying is merely a harmless “school prank”^(16, 17)^.

At the national level, the National Survey of School Health ‘PeNSE in Portuguese’ from 2009 to 2019 reported increasing rates of bullying victimization among Brazilian students, presenting the following data: 5.4% in 2009, 7.2% in 2012, 7.4% in 2015, and 23% in 2019. Recent studies also indicate concerning findings regarding symptoms exhibited by bullying victims, underscoring the need for family and school interventions not only for the victims but for the entire school community. This perspective is crucial in addressing the negative impacts associated with this phenomenon^(17, 18)^.

Given this context, and considering that they are recurrent targets of such violence within the current Brazilian social, political, and educational landscape, the community of individuals who deviate from cis-heteronormative models—especially LGBTQIAP+ adolescents—suffers from LGBT-phobic bullying, which is characterized by unequal power dynamics among peers. This type of bullying is often motivated by a trait that the aggressor deems a justification for violence, particularly in school environments, where educational practices should promote respect for diversity and actively combat intolerance and the unequal valuation of lives and bodies^(19, 20)^.

Furthermore, in 2018, Law No. 13.663 was enacted, incorporating the promotion of awareness, prevention, and actions to combat systematic intimidation and various forms of violence— especially bullying—into school policies. The law also emphasizes the importance of fostering a “culture of peace” within schools, recognizing the need for measures, actions, and interventions to address violent behaviors, humiliation, and discrimination against more vulnerable groups^(21)^.

Education, understood as a social practice that fosters gradual changes in ways of acting, thinking, and feeling, is increasingly utilized as a tool for facilitating learning and moderating the exchange of information. This is achieved through processes that encourage bonding and holistic support in knowledge construction^(22, 23, 24)^. In this study, particular emphasis is placed on the role of nurses as health educators^(25)^, especially within school environments and in their work with adolescents, aiming to enhance young people’s autonomy in promoting their own health and strengthening discussions on sexual and gender diversity, which require support and intervention in spaces dedicated to care.

Given the above, when analyzing the advancement of modern communication methods, it becomes evident that adolescent interactions have undergone a technological revolution. The rise of technology has introduced a new paradigm, in which internet access tools have been transformed, making it imperative to adopt new methodologies based on constructive approaches that depart from static and conservative teaching methods, which do not contribute to meaningful learning. Within this context, Digital Educational Technologies (DETs) have gained ground and now support learning processes through contemporary and innovative methodological perspectives^(26, 27, 28, 29)^.

At the same time, there is a notable lack of research focusing on LGBTQIAP+ adolescents, sexual and gender diversity, and, most importantly, the fight against LGBT-phobic bullying and the development of digital health interventions. This gap highlights the invisibility of young people from sexual and gender minorities and their vulnerabilities. The present study emerged in response to the scarcity of research on this subject, compounded by prejudice and discrimination within the academic community, which, due to a conservative and heteronormative viewpoint, has often silenced or neglected these topics, making them rarely discussed or explored in terms of research and publication interest in the LGBTQIAP+ field^(30, 31, 32)^.

This study is justified by the urgent need to develop new Information and Communication Technologies (ICTs) accessible to adolescents as a means of providing information, particularly within school settings, where sexual and gender diversity is a pressing, necessary, and inclusive topic of discussion. Thus, this study may contribute to the reduction of health risks faced by LGBTQIAP+ adolescents, which can be mitigated through knowledge dissemination.

## OBJECTIVES

To develop a Digital Educational Technology on LGBT-phobic bullying, in the form of a comic book, for health education among school-aged adolescents.

## METHODS

### Ethical Aspects

This research was conducted in accordance with the ethical principles established in Resolution No. 466, of December 12, 2012. The project was approved by the Research Ethics Committee of the Health Sciences Center at the Federal University of Pernambuco (UFPE). Consent for participation from individuals involved in the validation stages was obtained through the Informed Consent Form (ICF), signed by the judges participating in the content validation, and through the Assent Form, signed by school-aged adolescents in the appearance validation stage.

### Type of study

This is a methodological study aimed at developing a digital educational health technology, created between January and July 2023, in Recife, Pernambuco, Brazil, in comic book format for school-aged adolescents. The technology was subsequently validated in terms of content and appearance.

### Methodological procedures

Considering the stages of development for a DET, the PACO method (*Planning of Computer-Supported Learning Activities*) was used to guide the planning of computer-supported activities, taking into account pedagogical aspects and the characteristics of the target audience^(29, 32)^.

For this, the following methodological phases were followed: Selection of the objective, target audience, and theme; Organization; Selection of the theoretical framework; Design of instructional activities; Selection of digital tools to support activity implementation; Development of the digital resource; Evaluation^(29, 32)^.

Each phase was described to guide the systematic planning of instructional actions, ensuring their effective design and implementation. Phases 1 to 5 represent study stages, while Phases 6 and 7 correspond to the final outcomes of the technology development process.

### Study Stages

#### Phase 1: Selection of the objective, target audience, and theme

The initial steps proposed by the PACO method for developing a DET include: establishing the objective for creating this tool – to develop a digital educational technology within the context of sexual and gender diversity, in the form of a comic book, addressing LGBT-phobic bullying; defining the target audience for this technology – cis-heterosexual and LGBTQIAP+ school-aged adolescents; and selecting the theme/content to be included in this tool – LGBT-phobic bullying.

#### Phase 2: Organization of the educational comic book

This phase involves the organization of content, scriptwriting, and structuring of the technological tool. After defining the general objective, the topics to be addressed were identified, as well as the sequence and level of refinement in which the content would be presented, following a sequential learning logic^(31)^.

This stage was gradually refined as the technology planning progressed. To define and support the theoretical content, references from an integrative literature review were utilized. The purpose of this review was to analyze the development and use of educational technologies in health for adolescents and young LGBTQIAP+ individuals^(31)^. Additionally, scientific articles and official publications relevant to the study topic were reviewed.

**Figure 1:**
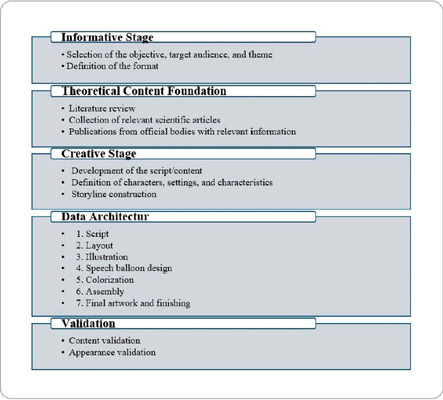
Stages of the structuring process for the technology in the format of an educational comic book, Recife, Pernambuco, Brazil, 2022

**Source:** *Adapted from Motta (2012), Lopes (2020), and Santos Júnior, Silva Júnior, and Souza Costa (2021).*

For the production of the comic book, methodological procedures were followed, incorporating necessary adaptations to meet the proposed objectives^(33, 34, 35)^. The stages followed are illustrated in Figure 1.

#### Phase 3: Selection of the pedagogical framework

For the development of the script, editing of the instructional content, and selection of the computational components of the DET, its creation was anchored in a previously selected pedagogical framework^(29)^. For this stage, the pedagogical framework of anthropologist, sociologist, and philosopher Edgar Morin was chosen, specifically his book *The Seven Complex Lessons in Education for the Future,* with an emphasis on Chapter Six, *Teaching Understanding,* under the perspective of integrating health education^(36)^ and fostering understanding and awareness in the context of sexual and gender diversity.

Morin’s approach served as an important theoretical foundation for the development of the technology, contributing to the creative aspects of the comic book, shaping the storyline and its key content elements, as well as influencing the character creation process. His conceptual approach was incorporated into the stages of narrative construction, scripting, and illustration, providing a reflective and awareness-building perspective in the storytelling of the comic book.

#### Phase 4: Design of the instructional actions in the educational comic book

For this phase, the selected theoretical framework, the profile of the target audience, and the requirements for the type of technology being developed and validated were considered^(29)^. Since this research involves the development of a tool with content produced from the initial stages of design, prior knowledge assessment, and opinion surveys, the structuring of these steps was built progressively as the early stages of this study were developed, allowing essential information to emerge for the production of the educational digital tool.

Morin’s theoretical framework influenced the design of the instructional actions in the comic book, incorporating elements aimed at educational purposes, emphasizing relationship-building and empathy while addressing the needs and uniqueness of individuals. This represents an educational model for the future, one that is taught through understanding, adopting a learning-facilitation perspective, and breaking away from conservative and static teaching models to construct meaningful learning experiences^(28, 36)^.

#### Phase 5: Digital tools to support the development of the educational comic book

To support the creation of the comic book, particularly regarding the data architecture stages—layout, illustration, speech balloon design, colorization, assembly, final artwork, and finishing—a graphic designer and illustrator was hired to execute the technical aspects of these steps using Procreate® and Adobe Photoshop®.

It is important to highlight that extensive research was conducted when selecting the graphic designer/illustrator, including analyses of various design styles and illustration characteristics to identify a comic book format that would best appeal to an adolescent audience^(34, 35)^.

As a result, the underground art style was chosen, heavily influenced by the artistic culture derived from Hip-Hop, featuring an organic aesthetic with elements of Brand-style illustrations, Motion Graphics, and 3D digital modeling. Additionally, during the hiring process, particular consideration was given to the professional’s extensive experience in graphic design, illustration, visual identity, and Street Art, as well as the fact that they are a member of the LGBTQIAP+ community, a crucial aspect in the creation of a technology focused on sexual and gender diversity^(37, 38)^.

## RESULTS

The comic book was titled “LGBT-Phobic Bullying: Shall We Talk?” and is intended for school-aged adolescents, with the goal of contributing to education and awareness about the prevention of LGBT-phobic bullying in the school environment. Additionally, it aims to promote, encourage, disseminate, and integrate this scientific production as a health educational technology product.

The storyline presents a narrative involving students, a teacher, and a healthcare professional, grounded in technical-scientific evidence, using simple and engaging language to foster reflection on the presence of LGBT-phobic bullying in schools. The main character, Diva, is a transgender adolescent. This technology addresses important topics such as respect, acceptance, equal opportunities, LGBT-phobic bullying, cyberbullying, identity groups, racism, and transphobia.

As previously demonstrated, the production of the comic book followed all the essential stages for constructing a graphic novel, namely: Scriptwriting; Layout; Illustration; Speech balloon design; Colorization; Assembly; Final artwork and finishing^(33, 34, 35)^.

During the layout, illustration, and speech balloon design stages, the story, panels, and characters began to take shape in accordance with the script, which included all the characteristics of each character^(34, 35)^.

**Figure 2:**
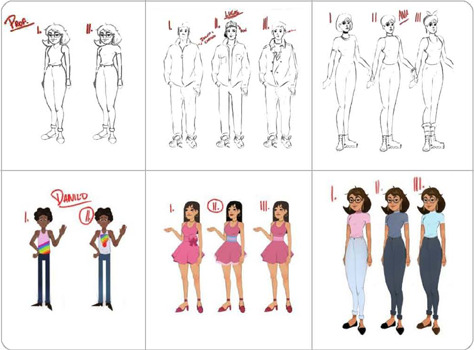
Character development ‘teacher, Lucas, Ana, Danilo, and Diva’ for the educational comic book LGBT-Phobic Bullying: Shall We Talk? – Drawing and colorization, Recife, Pernambuco, Brazil, 2023

**Figure 3:**
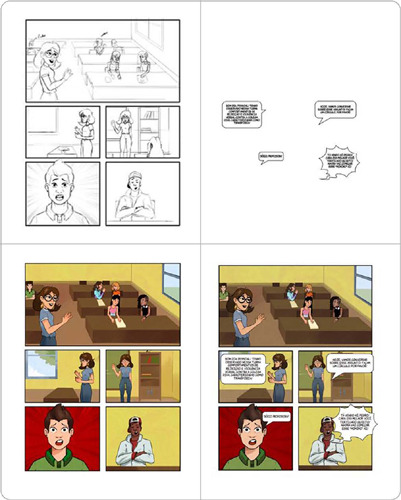
Stages of drawing, layout, speech balloon design, colorization, and assembly of page 5 from the educational comic book LGBT-Phobic Bullying: Shall We Talk?, Recife, Pernambuco, Brazil, 2023

Figures 2 and 3 illustrate these creative stages, which continued throughout the development of the comic book pages, from the character creation phase during illustration to the layout, speech balloon design, colorization, and structural assembly of the pages that make up this technology.

**Figure 4:**
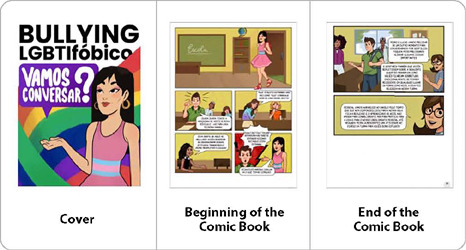
Final artwork of the educational comic book LGBT-Phobic Bullying: Shall We Talk? for the content validation stage, Recife, Pernambuco,

Additionally, the final artwork and finishing touches were completed with text revision and verification of the created material, as demonstrated in Figure 4, following the DET framework for content and appearance validation.

After its development, this educational comic book was validated in terms of content through the Educational Content Validation Instrument in Health^(39)^, by seven expert judges in the fields of adolescent health, health education, sexual and gender diversity, and LGBTQIAP+ health, selected according to Fehring’s criteria^(40)^.

**Figure 5:**
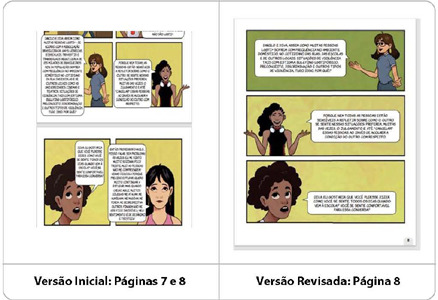
Illustrative representation of the comic book before and after structural adjustments: pagination, font size and speech balloons, increase in the number of pages, and revision of dialogues and panels (pages 7 and 8), Recife, Pernambuco, Brazil, 2024

At this stage, modifications were made based on the experts’ feedback, and all suggestions related to the structural aspects of the comic book were implemented, as illustrated in Figure 5. The technology underwent structural reformulation, including an increase in the number of pages, insertion of pagination, enhanced page layout, enlargement of font size and speech balloons, as well as text revision, with adjustments to some dialogues to make them shorter and less tiring to read.

During the appearance evaluation, 11 school-aged cis-heterosexual and LGBTQIAP+ adolescents assessed the educational comic book using the Educational Technology Appearance Validation Instrument, achieving an excellence standard in the Total Appearance Validity Index^(41)^.

## DISCUSSION

The educational comic book “LGBT-Phobic Bullying: Shall We Talk?” is an innovative digital technology, systematically developed with scientific methodological rigor, aimed at adolescents, contributing to the prevention of LGBT-phobic bullying in the school environment. All dialogues were carefully planned and collaboratively developed, including input from LGBTQIAP+ individuals, to determine the most effective way to discuss the topics addressed in the comic book. This approach fosters engagement and dialogue with the target audience in an educational, reflective, and awareness-raising manner.

Educational technologies serve as facilitators in the teaching-learning process and should be incorporated into health education practices, utilizing inclusive, engaging, and dialogue-driven communication to bring readers closer to the intended objectives, thereby facilitating knowledge assimilation^(42, 43)^.

Gaining increasing prominence, educational technologies have emerged with a transformative approach, integrating the fields of education and health communication to address challenges through scientifically based solutions, stimulating knowledge-building processes in a creative and engaging manner^(35, 44)^.

In this context, especially concerning adolescent audiences, the experience of narrated content combined with illustrations becomes a powerful tool for information dissemination and learning facilitation. The production of an educational comic book supports adolescent LGBTQIAP+ health promotion practices, strengthening discussions on sexual and gender diversity and preventing LGBT-phobic bullying in schools, where prejudice and negative attitudes reinforce stigma, exclusion, and violence.

Studies confirm that comic books are an engaging and accessible way to convey information, as they can simplify complex concepts, making them more comprehensible and appealing to audiences due to illustrations and engaging characters. This format makes the content more interactive, aiding readers in knowledge retention, particularly younger audiences, by stimulating imagination and learning^(45, 46)^.

eThis playful learning method can be particularly effective in addressing challenging and sensitive topics, where simple language and visual representations help overcome barriers, enabling readers to identify with the stories, thus facilitating message internalization and encouraging positive behaviors. Therefore, paying attention to details and understanding the target audience’s characteristics, especially the quality of illustrations, is crucial when designing educational materials for school-aged adolescents, as these aspects are essential for comprehension and engagement^(45, 46, 47)^.

Furthermore, regarding details, special emphasis is placed on the careful selection of a pedagogical framework, promoting the integration of health education and the concept of understanding within the context of sexual and gender diversity, based on Morin’s contributions. His influence was present throughout the stages of narrative and script development, the composition and characterization of the storyline, and the creation of certain characters, such as the teacher and the guest character, nurse Danilo, who serves as a mediator in classroom discussions, thus helping to structure the instructional actions and guidelines for the development of this DET^(36)^.

Additionally, from the initial concept development, scriptwriting, instructional content editing, and data architecture to the layout, illustration, speech balloon design, colorization, assembly, final artwork, and finishing stages, extensive research was conducted on graphic design styles and illustration characteristics to develop a comic book format that is engaging and appealing to adolescents^(34, 35, 38)^.

As a result, the underground art style was chosen, heavily influenced by Hip-Hop artistic culture, featuring Brand-style illustrations, Motion Graphics, and 3D digital modeling. The illustrations were created by an LGBTQIAP+ artist, a crucial factor in hiring the graphic designer, considering their expertise in accurately and organically portraying the characters.

After its development, validating the technology became essential to ensure the quality of the tool created, with the goal of determining whether the instrument accurately represents the phenomenon under investigation. This validation was conducted through expert judgment, providing scientific recognition of the aspects assessed in the technology analysis^(48)^. The comic book was validated by expert judges in the fields of adolescent health, health education, sexual and gender diversity, and LGBTQIAP+ health, ensuring that the content of this tool adequately reflected its intended construct, based on three-dimensional evaluation and levels of agreement deemed acceptable concerning the attributes of the developed technology.

Moreover, as important as the opinion of expert judges, another critical step in the development process was the appearance validation phase, which played a crucial role in constructing this tool. This step was essential because the legitimacy and credibility of the technology as an educational resource must also be reinforced by user evaluation, ensuring proper alignment between the material and its intended audience, thereby enhancing comprehension of the produced content^(42, 43, 49)^.

In summary, the creation of this tool, in conjunction with school-based health education practices, contributes to the maintenance or deconstruction of prejudices, promoting the development of value transformation processes from the perspective of cultural and dialogical psychology. This approach challenges vertical models of monologism and heteronomy, commonly found in educational settings and often reflected in social interactions and relationships^(50)^.

### Study Limitations

A key limitation of this study is the scarcity of previous research^(30, 31)^ on topics related to sexual and gender diversity, LGBT-phobic bullying, and the development of digital health innovation technologies aimed at LGBTQIAP+ adolescents. These are current and highly relevant issues that require greater attention and space within the academic and scientific community to advance research in the fields of health sciences, nursing, and adolescent LGBTQIAP+ health.

### Contributions to Nursing, Health, and Public Policy

The findings of this research are significant for advancing efforts to address exclusionary experiences and bullying faced by LGBTQ-IAP+ adolescents, particularly through the development of DET as a methodological strategy with pedagogical potential. This technology has the potential to play a crucial role in integrating health education processes, focusing on adolescent LGBTQIAP+ health and school nursing, particularly in preventing exclusion, prejudice, and violence. Additionally, it contributes to the strengthening of support networks, inclusion, and health promotion for LGBTQIAP+ adolescents.

This is a highly relevant and timely topic, warranting attention and space within the academic and scientific community, while also driving further research in the fields of health sciences, nursing, and adolescent LGBTQIAP+ health.

## FINAL CONSIDERATIONS

As a contemporary approach aimed at preventing LGBT-phobic bullying, promoting adolescent LGBTQIAP+ health, and reducing minority stress experienced daily by these individuals, the educational comic book “LGBT-Phobic Bullying: Shall We Talk?” was developed to address a sensitive topic in adolescent LGBTQIAP+ health in an engaging and reflective manner.

The combination of visual storytelling in comics with educational messages can effectively contribute, alongside educational practices, to reducing stigma, prejudice, discrimination, and violence in school environments against LGBTQIAP+ individuals, particularly benefiting school-aged adolescents.

Tools like this help make complex concepts and topics more comprehensible and appealing to the public, especially when targeted at school-aged adolescents. This type of approach enhances communication and interactivity, facilitating knowledge retention through a playful format, using simple and inclusive language with visual representations that help overcome social barriers and vulnerable contexts. Moreover, it promotes knowledge democratization, countering conventional texts and more traditional teaching approaches.

In conclusion, intervention studies are recommended to test the developed technology, ensuring its practical application among school-aged adolescents and validating its effectiveness as an essential tool for disseminating information on LGBT-phobic bullying prevention. Thus, this tool can help reduce health risks faced by LGBTQIAP+ adolescents, which can be mitigated through health education and the transformation of traditional teaching-learning processes, fostering knowledge-building through innovative and inclusive approaches.
