# Supplementary material for: Digital educational technology on LGBT-phobic bullying for school-aged adolescents
Source: Rev Bras Enferm. 2025 Sep 8;78(3):e20240377. doi: 10.1590/0034-7167-2024-0377 (PMC12419781; doi:10.1590/0034-7167-2024-0377)
Supplement: Translation

# Translation: Tecnologia educacional digital sobre *bullying* LGBTIfóbico para adolescentes escolares

**DOI:** 10.1590/0034-7167-2024-0377pt

**Authors:** Mariana Mercês Mesquita Espíndola, Ednaldo Cavalcante de Araújo, Adrian Thaís Cardoso Santos Gomes da Silva, Danilo Martins Roque Pereira, Marclineide Nóbrega de Andrade Ramalho, Thainara Torres de Oliveira, Weslla Karla Albuquerque Silva de Paula, Josueida de Carvalho Sousa

**Affiliations:** I Universidade Federal de Pernambuco. Recife, Pernambuco, Brasil; II Universidade Católica de Pernambuco. Recife, Pernambuco, Brasil

**Keywords:** Adolescente, Bullying, Minorias Sexuais e de Gênero, Revista em Quadrinhos, Tecnologia Educacional

## Abstract

**Objetivos:**

desenvolver uma tecnologia educacional digital sobre *bullying* LGBTIfóbico, no formato de gibi, para a educação em saúde de adolescentes escolares.

**Métodos:**

estudo metodológico utilizando o método de Planejamento de Atividades de Aprendizado Apoiadas por Computador para direcionar a organização das etapas de desenvolvimento, aliado ao referencial pedagógico de Edgar Morin, sob a ótica da compreensão, da educação em saúde e do contexto da diversidade sexual e de gênero.

**Resultados:**

o gibi “*Bullying LGBTIfóbico: vamos conversar?”* foi desenvolvido com o propósito de contribuir para a in(formação) no enfrentamento do *bullying* LGBTIfóbico no ambiente escolar, constituindo-se como um produto de tecnologia educacional em saúde.

**Considerações Finais:**

Como abordagem contemporânea, essa tecnologia foi desenvolvida para abordar de forma reflexiva um tema sensível à saúde do adolescente LGBTQIAP+, cuja combinação do poder narrativo-visual dos quadrinhos com mensagens educacionais poderá contribuir, junto às práticas educativas, para a redução do estigma, preconceito, *bullying* e outras formas de violência contra adolescentes LGBTQIAP+.

## INTRODUÇÃO

Adolescentes lésbicas, gays, bissexuais, travestis, transexuais, transgêneros, queer/questionando, intersexo, assexuais/arromântiques/agênero, poli/pansexual e outros pertencentes à diversidade sexual e de gênero (LGBTQIAP+), em sua maioria, estão inseridos em contextos de vulnerabilidade, nos quais, rotineiramente, são alvos de *bullying,* estigma, violência verbal, psicológica e/ou física, que podem culminar em suicídio e até assassinato perpetrado pelo agressor. Além disso, convivem com a discriminação social, que afeta sua qualidade de vida e bem-estar psicológico, causando-lhes sentimentos negativos, sintomas de ansiedade e outros comportamentos vulneráveis à saúde^(1, 2, 3)^.

Caracterizada por transformações físicas, psíquico-emocionais e sociais, por questionamentos e sentimentos de autoaceitação, é nessa fase, entre a adolescência e a juventude, que comportamentos e atitudes são desenvolvidos, e hábitos e valores são formados, influenciando o bem-estar e a evolução desses indivíduos^(4, 5, 6)^. Nesse sentido, as discussões acerca do exercício da sexualidade nesse período são importantes para a compreensão das vivências e experiências, cuja abordagem dessa temática, frequentemente, torna-se um tabu, com debates marcados por características superficiais e velados por preconceitos e constrangimentos. Isso ocorre em detrimento de cenários pseudo-normativos que, por vezes, desnaturalizam vivências que deveriam ser experienciadas como componentes elementares e integrativos à vida de cada ser humano^(7, 8)^.

Define-se identidade de gênero como a percepção que a pessoa tem de si, de forma individual e intrínseca, como sendo do sexo feminino, masculino ou uma combinação destes, podendo estar ou não em conformidade com seu sexo biológico. A expressão de gênero diz respeito à maneira como a pessoa se expressa, veste-se ou se apresenta, relacionando-se a aspectos de escolha na aparência, incluindo vestuário, modo de falar, linguagem corporal e formas de interação social com outras pessoas^(9, 10)^.

A terminologia “trans” é um “termo guarda-chuva” para se referir às pessoas que se identificam, no Brasil, como travestis, mulheres transexuais, homens trans, pessoas transmasculinas ou transfemininas, pessoas não binárias e demais indivíduos com diversidades de gênero. Para os adolescentes trans, os obstáculos a serem enfrentados são vivenciados desde o contexto da construção da sua identidade de gênero até as dimensões de aceitação familiar e socialização no ambiente escolar. Quando estigmatizados, esses adolescentes vivenciam situações de exclusão, discriminação, *bullying* LGBTIfóbico, insegurança e atitudes negativas que alicerçam cenários de violência^(2, 11, 12, 13, 14, 15)^.

Dito isso, o *bullying* no ambiente escolar caracteriza-se como uma das formas de violência e é um fenômeno mundial e complexo da contemporaneidade, que ocorre nas relações interpessoais, a partir de um constructo de dominação influenciado por diversos fatores (individuais, sociais, emocionais, psicológicos, contextuais etc.), com correlação direta com sintomas de depressão, ansiedade, estresse e ideação suicida, desmistificando a crença de que esse fenômeno se trata apenas de uma “brincadeira” escolar^(16, 17)^.

Em âmbito nacional, as edições da Pesquisa Nacional de Saúde Escolar (PeNSE), de 2009 a 2019, evidenciaram crescentes taxas de vitimização quanto ao *bullying* entre os brasileiros, apresentando os seguintes dados: 5,4% em 2009, 7,2% em 2012, 7,4% em 2015 e 23% em 2019. Estudos recentes também indicam dados preocupantes quanto aos sintomas apresentados pelas vítimas de *bullying,* evidenciando a necessidade de intervenção familiar e escolar não apenas para a vítima, mas para toda a comunidade escolar. Essa é uma perspectiva importante a ser considerada, em atenção aos impactos negativos que esse fenômeno ocasiona^(17, 18)^.

Em vista disso, e sendo recorrentes alvos dessas violências, considerando o atual contexto social, político e educacional brasileiro, a comunidade de pessoas dissidentes dos moldes cis-heteronormativos, sobretudo os adolescentes LGBTQIAP+, sofre com o *bullying* LGBTIfóbico característico das relações de poder desiguais entre pares, sendo motivado por alguma característica que o agressor considera passível de violência, principalmente no ambiente escolar, cujas práticas educativas devem corroborar com o respeito à diversidade e o enfrentamento da intolerância e da valoração desigual de vidas e corpos^(19, 20)^.

Outrossim, em 2018, foi promulgada a Lei nº 13.663, que inclui a promoção de medidas de conscientização, prevenção e combate à intimidação sistemática e a diversos tipos de violência, sobretudo o *bullying,* bem como a formação de uma “cultura de paz” entre as incumbências da escola, reconhecendo a importância de medidas, ações e intervenções para o enfrentamento de comportamentos violentos, atos de humilhação ou discriminação contra grupos mais vulneráveis^(21)^.

Compreendida como uma prática social que preconiza modificações graduais nos modos de agir, pensar e sentir, as estratégias de educação são utilizadas cada vez mais como instrumento facilitador da aprendizagem e moderador no compartilhamento de informações, sob a perspectiva dos processos de vínculo e acolhimento integral na construção do conhecimento^(22, 23, 24)^. Para este estudo, destaca-se a relevância dos enfermeiros e seu legítimo papel enquanto educadores em saúde^(25)^, sobretudo no ambiente escolar e no acompanhamento do público adolescente, buscando a autonomia desses jovens na promoção da própria saúde e no fortalecimento de questões em que a diversidade sexual e de gênero necessita de apoio e intervenção para mudanças nos espaços de cuidado.

Ante o exposto, ao analisar o avanço dos meios modernos de comunicação, percebe-se que os modos de interação entre os adolescentes passaram por uma revolução tecnológica, cuja ascensão da tecnologia evidenciou um novo paradigma, no qual as ferramentas de acesso à internet têm sido transformadas, tornando imperativa a adoção de novas metodologias, a partir de propostas construtivas, buscando romper com o ensino estático e conservador que não colabora para a promoção de uma aprendizagem significativa. Nesse cenário, as Tecnologias Digitais Educacionais (TEDs) ganham espaço e apoiam os processos de aprendizagem sob uma perspectiva metodológica contemporânea e inovadora^(26, 27, 28, 29)^.

Evidencia-se, oportunamente, a escassez de pesquisas que envolvam adolescentes LGBTQIAP+, a diversidade sexual e de gênero e, sobretudo, o enfrentamento ao *bullying* LGBTIfóbico e a construção de intervenções digitais em saúde, demonstrando a invisibilidade dos jovens de minorias sexuais e de gênero e suas vulnerabilidades. O presente estudo surgiu da escassez de pesquisas sobre esse tema, pesando-se, também, a presença de preconceito e discriminação por parte da comunidade acadêmica com visão conservadora e heterocisnormativa, que velam determinados temas, pouco discutidos e menos ainda explorados, no que tange ao interesse em estudos e publicações na área LGBTQIAP+^(30, 31, 32)^.

Este estudo justifica-se pela necessidade do desenvolvimento de novas Tecnologias de Informação e Comunicação (TICs) acessíveis aos adolescentes como meio de prover informação, sobretudo nos diversos contextos vivenciados no ambiente escolar, onde a diversidade sexual e de gênero se apresenta como uma discussão cogente, necessária e inclusiva. Assim, este estudo pode contribuir para a redução de agravos à saúde dos adolescentes LGBTQIAP+, os quais podem ser minimizados pela disseminação do conhecimento.

## OBJETIVOS

Desenvolver uma tecnologia educacional digital sobre *bullying* LGBTIfóbico, no formato de gibi, para educação em saúde de adolescentes escolares.

## MÉTODOS

### Aspectos éticos

A presente pesquisa foi conduzida em conformidade com os princípios éticos previstos na Resolução nº 466, de 12 de dezembro de 2012. O projeto foi aprovado pelo Comitê de Ética em Pesquisa (CEP) do Centro de Ciências da Saúde da Universidade Federal de Pernambuco (UFPE). O consentimento de participação dos indivíduos envolvidos nas etapas de validação foi obtido por meio do Termo de Consentimento Livre e Esclarecido (TCLE), assinado pelos juízes participantes da validação de conteúdo, e do Termo de Assentimento Livre e Esclarecido (TALE), assinado pelos adolescentes escolares na etapa de validação de aparência.

### Tipo de estudo

Trata-se de um estudo metodológico para o desenvolvimento de uma tecnologia digital educacional em saúde, construída no período de janeiro a julho de 2023, em Recife, Pernambuco, Brasil, no formato de gibi para adolescentes escolares, e posteriormente validada quanto ao conteúdo e à aparência.

### Procedimentos metodológicos

Considerando as etapas para o desenvolvimento de uma Tecnologia Digital Educacional (TDE), foi utilizado o método PACO: Planejamento de Atividades de Aprendizado Apoiadas por Computador, que tem como objetivo direcionar o planejamento de atividades apoiadas por computador, considerando os aspectos pedagógicos e as características do público-alvo^(29, 32)^.

Para isso, foram seguidas as seguintes fases metodológicas: Escolha do objetivo, público-alvo e tema; Organização; Escolha do referencial; Delineamento das ações instrucionais; Escolha das ferramentas digitais para apoio à realização das atividades; Construção do recurso digital; Avaliação^(29, 32)^.

Cada fase foi descrita de modo a orientar o planejamento ordenado das ações instrucionais construídas e executadas. As fases estruturais de 1 a 5 são descritas como etapas do trabalho. As fases 6 e 7 são resultados da construção dessa tecnologia.

### Etapas do trabalho

#### Fase 1) Escolha do objetivo, público-alvo e tema

Os passos iniciais propostos pelo método PACO na construção de uma TDE incluem: estabelecer o objetivo de criação dessa ferramenta – desenvolver uma tecnologia digital educacional no contexto da diversidade sexual e de gênero, no formato de gibi, sobre *bullying* LGBTIfóbico; definir o público-alvo ao qual essa tecnologia se destina – adolescentes escolares cis-heterossexuais e LGBTQIAP+; e escolher o tema/conteúdo a ser incluído nesta ferramenta – *bullying* LGBTIfóbico.

#### Fase 2) Organização do gibi educacional

Nesta fase, referente à organização do conteúdo, roteiro e composição da ferramenta tecnológica, após a definição do objetivo geral, foram designados os tópicos que seriam abordados, bem como a ordem e os níveis de refinamento com que os conteúdos seriam apresentados, seguindo uma lógica sequencial de aprendizagem^(31)^.

Essa etapa foi sendo delineada à medida que o planejamento da tecnologia avançava. Para a definição e embasamento do conteúdo teórico da tecnologia, foram utilizados referenciais provenientes de uma revisão integrativa da literatura previamente realizada, cujo objetivo foi analisar o desenvolvimento e o uso de tecnologias educacionais em saúde para adolescentes e jovens LGBTQIAP+^(31)^, além da pesquisa de artigos científicos pertinentes e publicações de órgãos oficiais com informações relevantes sobre a temática estudada.

Para a produção do gibi, foram seguidos procedimentos metodológicos com as adaptações necessárias ao cumprimento dos objetivos propostos^(33, 34, 35)^. As etapas seguidas estão demonstradas na Figura 1.

**Figura 1:** Etapas do processo de estruturação da tecnologia em formato de gibi educacional, Recife, Pernambuco, Brasil, 2022

*Fonte: Adaptado de Motta (2012), Lopes (2020) e Santos Júnior, Silva Júnior e Souza Costa (2021).*

#### Fase 3) Escolha do referencial pedagógico

Para a elaboração do roteiro, edição do conteúdo instrucional e seleção dos componentes computacionais da TDE, seu desenvolvimento foi ancorado em um referencial pedagógico previamente selecionado^(29)^. Para essa etapa, optou-se pelo referencial pedagógico do antropólogo, sociólogo e filósofo Edgar Morin, em seu livro “Os *sete saberes necessários à educação do futuro”,* com ênfase no capítulo seis dessa obra, *Ensinando a compreensão,* sob a ótica e articulação entre educação em saúde^(36)^ e a perspectiva da compreensão e sensibilização no contexto da diversidade sexual e de gênero.

Morin contribuiu como referencial importante no desenvolvimento da tecnologia, sendo utilizado na parte criativa do gibi, trazendo as características da história e seus principais aspectos em conteúdo e na criação dos personagens. Sua abordagem foi incorporada nas fases de construção do enredo, roteiro e desenho, proporcionando um olhar reflexivo e sensibilizador na contação da história em quadrinhos.

#### Fase 4) Delineamento das ações instrucionais do gibi educacional

Para essa fase, foram considerados o referencial escolhido, o perfil do público-alvo da pesquisa e as questões que envolviam o tipo de tecnologia a ser desenvolvida e validada^(29)^. Como esta pesquisa considera o desenvolvimento de uma ferramenta com conteúdo produzido a partir dos passos iniciais de desenvolvimento e aplicação de um instrumento de conhecimento prévio e opinião, o delineamento dessas etapas foi construído à medida que as fases iniciais deste estudo foram sendo produzidas, fazendo, assim, emergir informações imprescindíveis à produção da ferramenta tecnológica educacional.

O referencial de Morin trouxe ao delineamento das ações instrucionais do gibi características voltadas ao propósito educativo, sob a ótica do fortalecimento das relações e da empatia, com vistas às necessidades e singularidades do outro. Trata-se da educação para o futuro, ensinada por meio da compreensão, sob a perspectiva facilitadora da aprendizagem, rompendo com modelos de ensino conservadores e estáticos na construção de uma aprendizagem significativa^(28, 36)^.

#### Fase 5) Ferramentas digitais para apoiar a realização das atividades de construção do gibi educacional

Para apoiar a criação do gibi, no que diz respeito às etapas de arquitetura de dados – diagramação, desenho, balonamento, colorização, montagem, arte final e acabamento – foi necessária a contratação de um designer gráfico e ilustrador, que conduziu a execução técnica dessas etapas por meio dos softwares *Procreate®* e *Adobe Photoshop®.*

Ressalta-se que, na escolha do designer/ilustrador, foram realizadas diversas pesquisas sobre os tipos gráficos dos designs e as características das ilustrações, de modo a contemplar um tipo de história em quadrinhos que se encaixasse melhor ao público adolescente^(34, 35)^.

Por isso, optou-se pela arte underground, com forte influência da cultura artística derivada do Hip-Hop, em um estilo orgânico e com características de ilustrações do tipo *Brand, Motion Graphics* e modelagem 3D digital colorida. Ademais, na contratação do profissional, levou-se em consideração sua vasta experiência em design gráfico, ilustração, identidade visual e *Street Art,* além do fato de se tratar de uma pessoa LGBTQIAP+, aspecto relevante na criação de uma tecnologia sobre diversidade sexual e de gênero^(37, 38)^.

## RESULTADOS

O gibi foi intitulado “*Bullying LGBTIfóbico: vamos conversar?”* e é destinado a adolescentes escolares, com o objetivo de contribuir com a in(formação) sobre a prevenção do *bullying* LGBTIfóbico no ambiente escolar, bem como promover, incentivar, divulgar e socializar essa produção científica como um produto de tecnologia educacional em saúde.

O enredo apresenta uma história entre estudantes, uma professora e um profissional da saúde, contextualizada com fundamentação técnico-científica, em uma linguagem simples e descontraída, a fim de favorecer a reflexão sobre a presença do *bullying* LGBTIfóbico na escola. A personagem principal, Diva, é uma adolescente transexual. Na tecnologia, são discutidos temas importantes, como respeito, aceitação e igualdade de condições, *bullying* LGBTIfóbico, *cyberbullying,* grupos identitários, racismo e transfobia.

Conforme demonstrado anteriormente, a produção do gibi percorreu todas as etapas essenciais para a construção de uma história em quadrinhos, a saber: 1. Roteiro; 2. Diagramação; 3. Desenho; 4. Balonamento; 5.Colorização; 6. Montagem; 7. Arte final e acabamento^(33, 34, 35)^.

Nas etapas de diagramação, desenho e balonamento, a história, os quadrinhos e os personagens começaram a tomar forma, de acordo com o disposto no roteiro, no qual foram incluídas todas as características de cada personagem^(34, 35)^.

A seguir, as Figuras 2 e 3 ilustram essas etapas criativas, que deram continuidade à construção das páginas do gibi, desde a criação dos personagens, na fase de desenho, até a diagramação, balonamento, colorização e montagem estrutural das páginas que compuseram essa tecnologia.

**Figura 2:** Construção dos personagens (professora, Lucas, Ana, Danilo e Diva) do gibi educacional: *Bullying* LGBTIfóbico: Vamos conversar? - Desenho e colorização, Recife, Pernambuco, Brasil, 2023

**Figura 3:** Etapas de desenho, diagramação, balonamento, colorização e montagem da página 5 do gibi educacional - *Bullying* LGBTIfóbico: Vamos conversar?, Recife, Pernambuco, Brasil, 2023

Ademais, a arte final e os acabamentos foram realizados com revisão do texto e conferência do material criado, conforme demonstrado na Figura 4, seguindo a TDE para respectivas validações de conteúdo e aparência.

**Figura 4:** Arte final de gibi educacional: *Bullying* LGBTIfóbico: vamos conversar? para etapa de validação de conteúdo, Recife, Pernambuco, Brasil, 2023

Após o seu desenvolvimento, esse gibi educacional foi validado quanto ao conteúdo por meio do Instrumento de Validação de Conteúdo Educacional em Saúde (IVCES)^(39)^, por sete juízes especialistas nas áreas de saúde do adolescente, educação em saúde, diversidade sexual e de gênero e saúde LGBTQIAP+, selecionados de acordo com os critérios de Fehring^(40)^.

Nesta etapa, foram realizadas alterações com base nas considerações dos especialistas, e todas as sugestões relacionadas à parte estrutural do gibi foram acatadas, conforme representação ilustrativa demonstrada na Figura 5. Houve uma reformulação estrutural da tecnologia, com aumento no número de páginas, inserção da paginação, ampliação do preenchimento, aumento do tamanho da fonte e dos balões, além da revisão do texto, com a reformulação de algumas falas para torná-las menores e menos cansativas para a leitura.

**Figura 5:** Representação ilustrativa do Gibi, antes e depois de ajustes estruturais: paginação, tamanho da fonte e dos balões, ampliação do número de páginas e remodelação de falas e quadrinhos, páginas 7 e 8, Recife, Pernambuco, Brasil, 2024

Na avaliação de aparência, 11 adolescentes escolares cis heterossexuais e LGBTQIAP+ julgaram o Gibi educacional através do Instrumento de Validação de Aparência de Tecnologia Educacional em Saúde (IVATES) com padrão de excelência quanto ao Índice de Validade de Aparência Total (IVA-T)^(41)^.

## DISCUSSÃO

O gibi educacional “*Bullying LGBTIfóbico: vamos conversar?”* é uma tecnologia digital inovadora, construída de modo sistemático e com rigor metodológico científico, voltada para adolescentes, coadjuvando na prevenção do *bullying* LGBTIfóbico no ambiente escolar. Todos os diálogos foram pensados e elaborados em conjunto, inclusive por pessoas LGBTQIAP+, a fim de definir a melhor forma de discutir os temas abordados no gibi, promovendo aproximação e diálogo com o público-alvo, em um caráter educativo-reflexivo e sensibilizador.

As tecnologias educacionais são instrumentos facilitadores no processo de ensino-aprendizagem e devem ser inseridas nas práticas de educação em saúde, utilizando discursos e uma linguagem acolhedora e dialógica, que aproxime o leitor/público do objetivo proposto, facilitando, assim, a assimilação dos conhecimentos^(42, 43)^.

Cada vez mais em evidência, as tecnologias educacionais têm emergido com um enfoque transformador, interligando o campo da educação com a comunicação na área da saúde para a solução de problemas, com uso de fundamentação científica, estimulando os processos de construção do conhecimento de modo criativo e atrativo^(35, 44)^.

Nesse cenário, sobretudo no que se refere ao público adolescente, a experiência do contexto narrado com ilustrações surge como uma ferramenta informativa e moderadora nos processos de ensino-aprendizagem. A produção de um gibi educacional colabora para as práticas de promoção da saúde do adolescente LGBTQIAP+, fortalecendo as questões que envolvem a diversidade sexual e de gênero e a prevenção do *bullying* LGBTIfóbico no ambiente escolar, cujo preconceito e atitudes negativas alicerçam cenários de estigma, exclusão e violência.

Estudos comprovam que as leituras em quadrinhos são uma forma cativante e acessível de transmitir informações, uma vez que possuem a capacidade de simplificar conceitos complexos, tornando-os mais compreensíveis e atraentes para o público, devido à presença das ilustrações e dos personagens envolventes. Isso torna o conteúdo mais interativo e auxilia o leitor na assimilação das informações, especialmente para o público juvenil, instigando a imaginação e a aprendizagem^(45, 46)^.

Ademais, ilustrações coloridas, balões com falas e personagens interessantes, presentes nas histórias em quadrinhos, facilitam a retenção da informação, sobretudo no ambiente escolar, auxiliando na promoção da saúde dos adolescentes e oportunizando a mudança de hábitos danosos à saúde, com vistas à melhoria da qualidade de vida desses indivíduos^(45, 46, 47)^.

Esse método lúdico de aprendizado pode ser particularmente eficaz para abordar temáticas desafiadoras e sensíveis, nas quais a linguagem simples e as representações visuais ajudam a superar barreiras, permitindo que os leitores se identifiquem com as histórias, facilitando a internalização das mensagens e incentivando a adoção de comportamentos positivos. Por conseguinte, pensar nos detalhes e nas características do público receptor da mensagem, sobretudo na qualidade do desenho, torna-se fundamental quando se trata do perfil do adolescente escolar, uma vez que tais aspectos são imprescindíveis para a compreensão e estimulação do leitor juvenil^(45, 46, 47)^.

Ademais, em se tratando de detalhes, ressalta-se a minuciosa escolha de um referencial pedagógico, promovendo a articulação entre educação em saúde e a perspectiva da compreensão no contexto da diversidade sexual e de gênero, com base em Morin. Suas contribuições se deram desde as fases de construção do enredo e roteiro, passando pela composição e caracterização da história, até a concepção de alguns personagens, como a professora e o personagem convidado e mediador da discussão em sala de aula, o enfermeiro Danilo, auxiliando, assim, no delineamento das ações e comandos de instrução para a produção desta TDE^(36)^.

Além disso, desde a concepção da ideia, elaboração do roteiro, edição do conteúdo instrucional e arquitetura de dados, até as fases de diagramação, desenho, balonamento, colorização, montagem, arte-final e acabamento, foram realizadas diversas pesquisas sobre os tipos gráficos dos designs e as características das ilustrações, a fim de contemplar um tipo de história em quadrinhos que fosse estimulante e atraente para os adolescentes^(34, 35, 38)^.

Optou-se, então, pelo design de arte modelo *underground,* com forte influência da cultura artística do Hip-Hop e características de ilustrações do tipo *Brand, Motion Graphics* e modelagem 3D digital colorida. A ilustração foi realizada por uma pessoa LGB-TQIAP+, característica importante na contratação do designer gráfico, visto sua expertise na retratação dos personagens de forma minuciosa e orgânica.

Após seu desenvolvimento, a validação da tecnologia tornou-se um elemento essencial para garantir a qualidade da ferramenta criada, a fim de determinar se o instrumento produzido representa adequadamente o fenômeno investigado, por meio do julgamento de especialistas, conferindo reconhecimento científico aos aspectos mensurados na análise da tecnologia^(48)^. Este gibi foi devidamente validado por juízes com expertise nas áreas de saúde do adolescente, educação em saúde, diversidade sexual e de gênero e saúde LGBTQIAP+, de modo a aferir adequadamente, em três dimensões, se o conteúdo desta ferramenta refletia seu constructo, por meio dos níveis de concordância julgados como aprovados em relação aos atributos da tecnologia desenvolvida.

Ademais, tão importante quanto a opinião dos juízes especialistas, outra etapa trabalhada com afinco foi a validação de aparência, fase essencial no processo de construção dessa ferramenta. Isso se deve ao fato de que a legitimidade e credibilidade da tecnologia a ser utilizada como fonte de orientação também devem estar alicerçadas na avaliação dos próprios usuários, garantindo, nesse processo, uma aproximação adequada entre o material e o público receptor, colaborando para a compreensão do conteúdo produzido^(42, 43, 49)^.

Em suma, a criação dessa ferramenta, em conjunto com as práticas de educação em saúde na escola, contribui para a manutenção ou a desconstrução de preconceitos, promovendo o desenvolvimento de processos de transformação de valores sob a perspectiva da psicologia cultural e dialógica, em contraposição a posturas verticais de monologismo e heteronomia, comumente encontradas nos cenários educacionais e, por vezes, expressas nas relações e interações sociais^(50)^.

### Limitações do estudo

Como limitações do estudo, aponta-se a escassez de pesquisas anteriores^(30, 31)^ sobre temas que envolvam a diversidade sexual e de gênero, o *bullying* LGBTIfóbico e o desenvolvimento de tecnologias nas áreas de inovação digital em saúde voltadas para adolescentes LGBTQIAP+. Esses são assuntos atuais e de grande relevância, que merecem atenção e espaço na comunidade acadêmica e científica para o avanço das pesquisas em ciências da saúde, enfermagem e saúde do adolescente LGBTQIAP+.

### Contribuições para a área da enfermagem, saúde ou política pública

Os resultados desta pesquisa são relevantes para o avanço no enfrentamento às vivências excludentes e ao *bullying* sofrido pelos adolescentes LGBTQIAP+, considerando o desenvolvimento de uma tecnologia educacional digital como uma estratégia metodológica com potencial pedagógico. Essa tecnologia pode desempenhar um papel crucial ao integrar processos de educação em saúde voltados à saúde do adolescente LGBTQIAP+ e à atuação da enfermagem escolar, no que tange à prevenção de situações de exclusão, preconceitos e até mesmo violências. Além disso, contribui para o fortalecimento do apoio, acolhimento e promoção da saúde dos adolescentes LGBTQIAP+. Trata-se de um tema atual e de grande relevância, que merece atenção e espaço na comunidade acadêmica e científica, além de impulsionar o avanço de pesquisas nas áreas de ciências da saúde, enfermagem e saúde do adolescente LGBTQIAP+.

## CONSIDERAÇÕES FINAIS

Como uma abordagem contemporânea voltada à prevenção do *bullying* LGBTIfóbico, à promoção da saúde de adolescentes LGBTQIAP+ e à redução do estresse de minorias vivenciado diariamente por essas pessoas, o gibi educacional “*Bullying LGBTIfóbico: vamos conversar?”* foi desenvolvido para abordar de forma cativante e reflexiva um tema sensível à saúde do adolescente LGBTQIAP+. A combinação do poder narrativo visual dos quadrinhos com mensagens educacionais poderá contribuir efetivamente, junto às práticas educativas, para a redução do estigma, preconceito, discriminação e violência no contexto escolar contra pessoas LGBTQIAP+, alcançando, sobretudo, os adolescentes escolares.

Ferramentas como essa tornam conceitos e temas complexos mais compreensíveis e atraentes ao público, especialmente quando se trata de adolescentes escolares. Esse tipo de abordagem torna o conteúdo mais comunicativo e interativo, facilitando a retenção de informações de modo lúdico, por meio de uma linguagem simples e acolhedora, com representações visuais que ajudam a superar barreiras sociais e contextos vulneráveis. Além disso, promove a democratização do conhecimento, contrapondo-se aos textos convencionais e às abordagens mais tradicionalistas.

Em conclusão, recomendam-se estudos de intervenção para testagem da tecnologia desenvolvida, de modo a colocá-la em prática com adolescentes escolares, possibilitando a comprovação de sua eficácia como um importante recurso na disseminação de informações sobre a prevenção do *bullying* LGBTIfóbico. Dessa forma, essa ferramenta pode auxiliar na redução de agravos à saúde dos adolescentes LGBTQIAP+, que podem ser minimizados por meio da educação em saúde e da transformação dos processos de ensino-aprendizagem conservadores, promovendo a construção de conhecimentos com abordagens inovadoras e inclusivas.
